# A Metabolic Model of Intestinal Secretions: The Link between Human Microbiota and Colorectal Cancer Progression

**DOI:** 10.3390/metabo11070456

**Published:** 2021-07-15

**Authors:** Pejman Salahshouri, Modjtaba Emadi-Baygi, Mahdi Jalili, Faiz M. Khan, Olaf Wolkenhauer, Ali Salehzadeh-Yazdi

**Affiliations:** 1 Department of Genetics, Faculty of Basic Sciences, Shahrekord University, Shahrekord 8818634141, Iran; pejmansalahshouri@gmail.com (P.S.); emadi-m@sku.ac.ir (M.E.-B.); 2 Biotechnology Research Institute, Shahrekord University, Shahrekord 8818634141, Iran; 3 Hematology, Oncology and SCT Research Center, Tehran University of Medical Sciences, Tehran 14114, Iran; mahjalili@sina.tums.ac.ir; 4 Department of Systems Biology and Bioinformatics, University of Rostock, 18051 Rostock, Germany; faiz.khan3@uni-rostock.de (F.M.K.); olaf.wolkenhauer@uni-rostock.de (O.W.)

**Keywords:** microbiome, genome-scale metabolic model, community metabolic modeling, colorectal cancer

## Abstract

The human gut microbiota plays a dual key role in maintaining human health or inducing disorders, for example, obesity, type 2 diabetes, and cancers such as colorectal cancer (CRC). High-throughput data analysis, such as metagenomics and metabolomics, have shown the diverse effects of alterations in dynamic bacterial populations on the initiation and progression of colorectal cancer. However, it is well established that microbiome and human cells constantly influence each other, so it is not appropriate to study them independently. Genome-scale metabolic modeling is a well-established mathematical framework that describes the dynamic behavior of these two axes at the system level. In this study, we created community microbiome models of three conditions during colorectal cancer progression, including carcinoma, adenoma and health status, and showed how changes in the microbial population influence intestinal secretions. Conclusively, our findings showed that alterations in the gut microbiome might provoke mutations and transform adenomas into carcinomas. These alterations include the secretion of mutagenic metabolites such as H_2_S, NO compounds, spermidine and TMA (trimethylamine), as well as the reduction of butyrate. Furthermore, we found that the colorectal cancer microbiome can promote inflammation, cancer progression (e.g., angiogenesis) and cancer prevention (e.g., apoptosis) by increasing and decreasing certain metabolites such as histamine, glutamine and pyruvate. Thus, modulating the gut microbiome could be a promising strategy for the prevention and treatment of CRC.

## 1. Introduction

Cancer is generally known as a disease of the genome arising out of a combination of genetic mutations, epigenetic modifications and altered signaling pathways. These mutations occur diversely, sometimes with undetermined origins. However, some cancers are associated with infectious agents, and some appear in tissues that are exposed to microbiota (a set of microbial agents present in a specific environment) [1]. 

Microbial agents constitute about 90% of the cells in the human body, and it is estimated that there are 10^14^ bacteria, comprising 10^3^ species, in the human colon. This signifies that bacterial genes outnumber human genes in the human body [2,3,4,5]. Additionally, the density of large intestinal bacteria is approximately 1010 times higher than that of the small intestinal bacteria, and the risk of cancer in the large intestine is 12 times higher than that that in the small intestine [6,7]. 

The transformation of adenoma into carcinoma tumors in colorectal cancer (CRC) requires mutations in the cancer-driver genes, which normally takes up to 10 years, and additional mutations accelerate its progression. There is also a hypothesis that considers the role of the microbiome in CRC, mostly adenomas. The driver–passenger hypothesis states that driver bacteria cause this transformation by triggering DNA damage and persistent inflammation. On the other hand, the tumor microenvironment provides another growth site for opportunistic bacteria called passengers. This even suggests that some probiotic bacteria may take advantage of the tumor microenvironment and prevent the progression of cancer [8].

With the advent of high-throughput technologies, researchers can quantify molecular changes at different cellular levels. These technologies can reveal the complete picture of cell metabolic activity in one snapshot and can unveil the metabolic patterns and function of cells, including the activities of enzymes, gene products, transporters and chemical reactions. Therefore, they would be suitable tools for the metabolic profiling of different cells, both to identify differences between organs and to distinguish metabolic diseases [9,10,11,12,13].

Genome-scale metabolic models (GEMs) provide quantitative information about the metabolism of large-scale systems [14]. Using GEMs and optimization methods, such as flux balance analysis (FBA) or flux variable analysis (FVA), the metabolic flux rate for all the reactions in the model can be predicted [15,16]. For deciphering the role of the entire system and the complex relationships of a microbial community, community metabolic modeling (CMM) is introduced [17]. Briefly, CMM is the combination of the ecological model of the microbiota (presence in an environment) and their microbiota GEMs [18]. CMM can employ GEMs to examine the interactions between different microorganisms and the cross-feeding in a population (the exchange of metabolites among microorganisms). Different studies have assessed the effect of cross-feeding between host human cells and environmental absorption using CMM. For example, Kumar et al. reconstructed CMM of malnourished children’s gut microbiota by the integration of GEMs. This model revealed a reduction in essential amino acid production by the gut microbiota in malnourished children [19]. Another study investigated metabolic alterations following changes to the gut microbiota composition in metformin-treated type 2 diabetes patients [20]. They suggested that lipopolysaccharide synthesis, nucleotide sugar metabolism and amino acid metabolism are susceptible to changes in gut microbes. The intestinal microbiota and metabolic changes in CRC have been studied using metagenomics and metabolic data, respectively [21,22]. Researchers have proposed some tumor-specific bacterial populations and metabolite regulation in CRC. Kehan Xu et al. investigated the co-occurrence and co-exclusion of bacterial species in the mucosa-associated microbiota of CRC tumors and found potential bacterial biomarkers in the CRC tumor’s microbiota [23].

Additionally, previous studies have explored changes in the bacterial population and their potential impact on CRC initiation or development, as well as metabolic alterations in the CRC tumor lumen and blood serum. By using the same modeling approach, they highlighted the role of *Fusobacterium* spp. in the production of glutarate and in the suppression of butyrate and acetate levels in feces [23,24]. Since then, many high-throughput data have unraveled the etiology and complexity of CRC; however, the investigation of CRC and microbiome metabolism remain a subject of inquiry. In the current study, we evaluated the CMM of the gut microbiome and its influence on the initiation and progression of CRC with a comprehensive and metabolomic approach. Our results indicate that alterations in the gut microbiome might provoke CRC’s transformation from an adenoma into a carcinoma.

## 2. Results

### 2.1. Community Microbiome Metabolic Models of CRC in Different Tumors

The community modeling of the microbiome requires the integration of individual bacterial metabolic models (GEMs) based on the proportions of different bacteria present through ecological modeling. In this work, we made use of the MGYS00001248 data [25], an ecological model that consists of metagenomic data of mucus biopsy specimens with adenomas and their adjacent tissue, as well as non-tumor tissue as a control [21]. Adjacent tissue samples of adenomas and carcinomas were discarded on the assumption that we did not know whether they were directly related to CRC (Table 1).

To select the GEMs’ best matching with existing metagenome data, we firstly tried to combine both publicly available databases, AGORA and EMBL. In 2016, AGORA models (consisting of 773 GEMs of well-known bacteria) were reconstructed to investigate the reciprocal association of bacterial behaviors and human metabolic diseases [26]. The AGORA developers showed that the interaction of the species depends on the availability of nutrients in the diet and the metabolic potential of the models. EMBL–GEMs were used to create and store automated GEMs of 5587 organisms at the strain level [27]. The AGORA models were compatible with the COBRA toolbox, but we found that the EMBL–GEMs were not. Furthermore, the EMBL–GEMs are insufficient in requisite models for the creation of community models. Since metagenomic data, which consist of bacteria’s taxonomy and their measure of presence, have insufficient resolution regarding the strains or species of bacteria, we should modify the taxon list based on experimental knowledge. Additionally, metagenomic data vary in depth and sample size, which could lead to missing data. Matching GEMs with bacteria requires identification at the strain or species level. To minimize the loss of the accuracy of microbial population information, pan-AGORA models (Supplementary File S1) were constructed using the COBRA toolbox, which are infrastructure models of subclasses of the family, genus and species models. Then, to estimate low-resolution reads of taxonomy assignment data, we used publications and the Disbiome database [28]. Thus, we created a table (Supplementary File S2) of bacterial GEMs’ names and their abundance for further analysis. 

**Figure 1:**
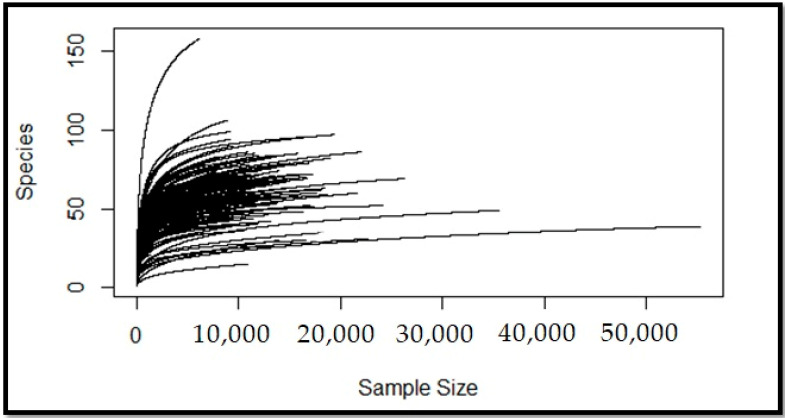
Rarefaction curves: the relations between the number of species present and total reads in each sample. We used them to perceive and select an appropriate sample size for normalization. As shown, most of the samples were about 10,000 or larger. Furthermore, at sample size n = 10,000, most of the curves are in asymptotes, which indicates that most of the species are present at this size.

Although using the rarefaction method for metagenomic data analysis is controversial [29,30,31], it seems rational for metagenomic data normalization [31]. However, one of the challenges with this method is choosing the right threshold value. In general, an appropriate value is one in which most of the samples are larger [32]. In the same way, the rarefaction curves can provide an estimate of the asymptotic richness concerning the sample counts that are suitable for normalization (Figure 1). By considering these two approaches, we selected a sample size of 10,000 for normalization. Therefore, samples with read counts below this value were discarded and, then, the abundances were normalized. Finally, the taxonomy assignment data were modified and normalized for input data from 864 read rows corresponding to 219 bacterial GEMs collected as the microbiome modeling toolbox input data (Supplementary File S2). 

Since diet can also affect the survival of bacterial models in the community model, we postulated that the environmental conditions of the microbial population were food-intensive, so all the simulations were performed under the rich diet. Finally, from 160 mucosal microbiome samples, 88 microbiome community models were constructed based on AGORA and Pan models (Supplementary File S3).

The NMPC (net maximal production capability), which simulates the capacity of the microbiota to create the intestinal lumen environment, was calculated by FVA (Supplementary File S4). This simulation was utilized to analyze the differences in the effects of the microbiome in each group on the metabolic conditions around the tumor and intestine. We consider the NMPC as the microbiome’s role in creating the intestinal environment.

### 2.2. Meta-Model Selection and Data Analysis for Simulated Metabolism of CRC Microbiome 

**Figure 2:**
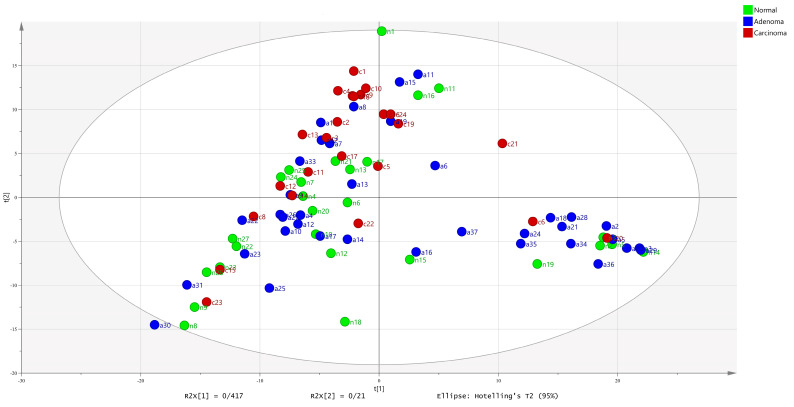
Scatter plot of PCA scores for all models. Using PCA, meta-models were selected for each group by considering the greatest similarity in groups (nearest) and the greatest differences between groups (farthest). a3, a5, a20, a21, a28, a29, a34 and a36 as an adenoma meta-model; c1, c7, c9, c10, c16, c18 and c24 as a carcinoma meta-model; and n8, n9, n22, n23, n26 and n27 as a normal meta-model. In this figure, a, c and n stand for adenoma, carcinoma and normal, respectively. This PCA was plotted by the first two components, in which R2X[1] = 0.417 and R2X[2] = 0.21.

Studies have demonstrated that the CRC microbiota and its healthy counterpart have diverse patterns [21], which are called a meta-community. Accordingly, we call models with the same patterns a meta-model. Thus, we first detected the meta-models of all the groups based on the NMPC data and then selected a meta-model for each group (n8, n9, n22, n23, n26 and n27 as a normal meta-model; a3, a5, a20, a21, a28, a29, a34 and a36 as an adenoma meta-model; and c1, c7, c9, c10, c16, c18 and c24 as a carcinoma meta-model). This step was performed by PCA (Figure 2). The PCA plots show the similarity of the microbiome metabolic models based on their NMPCs according to the first two components. Presumably, microbiome metabolic models with the greatest similarity in NMPC data have the same patterns. We selected meta-models with the most similarity within groups and the most dissimilarity between groups. Cross-validation with the SIMCA software indicated the significance of PCA models, with R2X(cum) = 0.85, and Q2(cum) = 0.727. Supplementary File S5 shows more statistical tests for this model.

### 2.3. Meta-Models Reveal Different Patterns Among CRC Tumors

**Figure 3:**
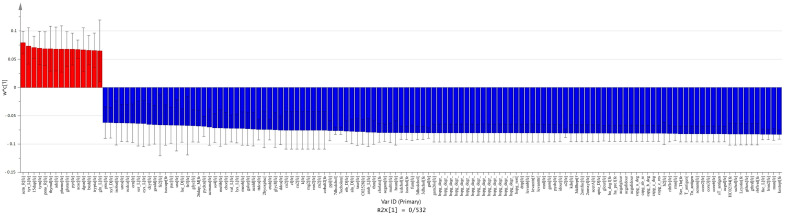
Extraction of the most important metabolites in carcinoma vs. normal meta-model comparison. The column plot of metabolites excluded by the VIP plot shows which metabolite correlates with which group. Red columns (positive w*c) are metabolites more abundant in carcinoma, and blue columns (negative w*c) are those more abundant in the normal group. These w*c measures are from the first component of the PLS-DA model. For this PLS-DA model, the first component R2 and Q2 parameters were R2X = 0.532 and Q2 = 0.972.

**Figure 4:**
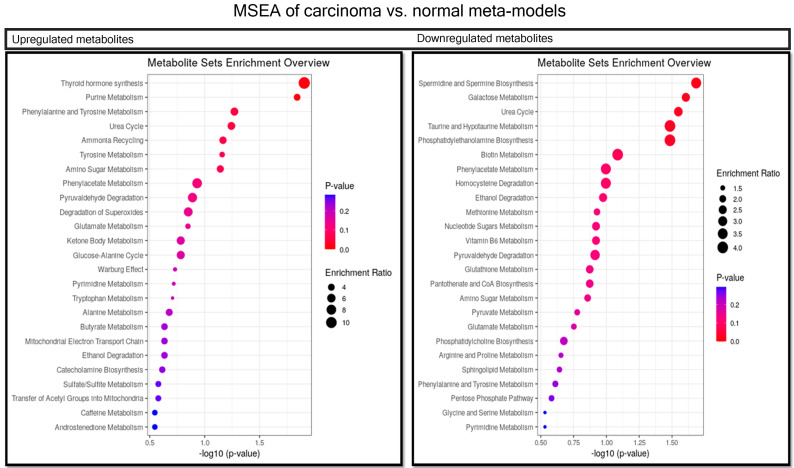
MSEA of the most important metabolites in PLS model of carcinoma vs. normal. Metabolites more abundant in carcinoma meta-models than normal meta-models involved in pathways are shown in the left-side table. Those more abundant in normal are shown in the right-side table. We considered *p*-values < 0.05 significant for this analysis.

**Figure 5:**
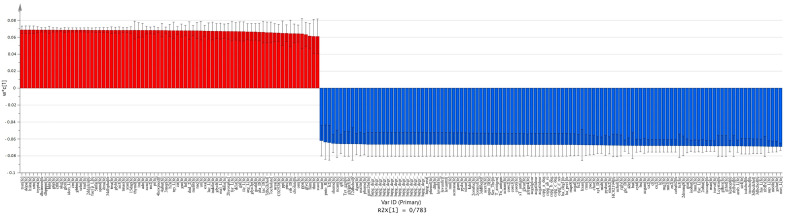
Extraction of most important metabolites in adenoma vs. normal meta-model comparison. The column plot of metabolites excluded by the VIP plot shows which metabolite correlates with which group. Red columns (positive w*c) are metabolites more abundant in adenoma, and blue columns (negative w*c) are those more abundant in the normal group. These w*c measures are from the first component of the PLS-DA model. For this PLS-DA model, the first component R2 and Q2 parameters were R2X = 0.783 and Q2 = 0.976.

**Figure 6:**
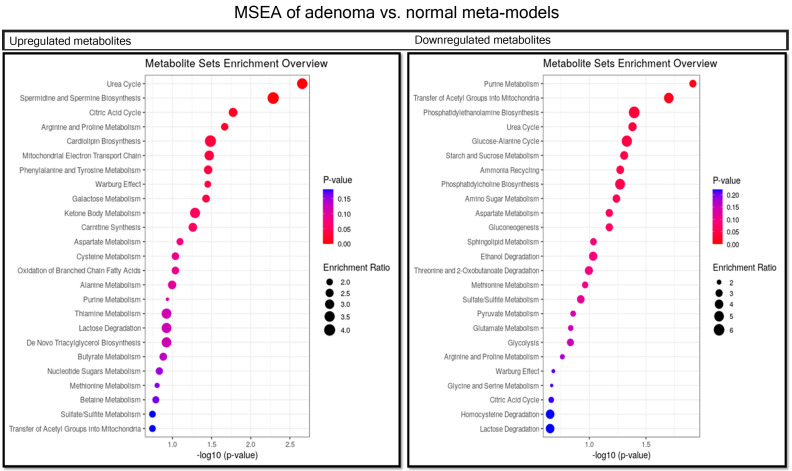
MSEA of the most important metabolites in PLS-DA model of adenoma vs. normal. Metabolites more abundant in the adenoma meta-model than the normal meta-model involved in pathways are shown in the left-side table. Those more abundant in normal are shown in the right-side table. We considered *p*-values < 0.05 significant for this analysis.

**Figure 7:**
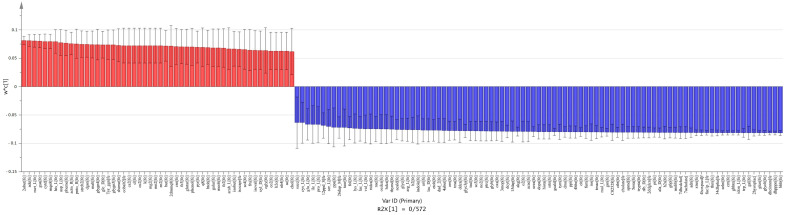
Extraction of the most important metabolites in carcinoma vs. adenoma meta-model comparison. The column plot of metabolites excluded by the VIP plot shows which metabolite correlates with which group. Red columns (positive w*c) are metabolites more abundant in carcinoma, and blue columns (negative w*c) are those more abundant in the adenoma group. These w*c measures are from the first component of the PLS-DA model. For this PLS-DA model, the first component R2 and Q2 parameters were R2X = 0.572 and Q2 = 0.933.

**Figure 8:**
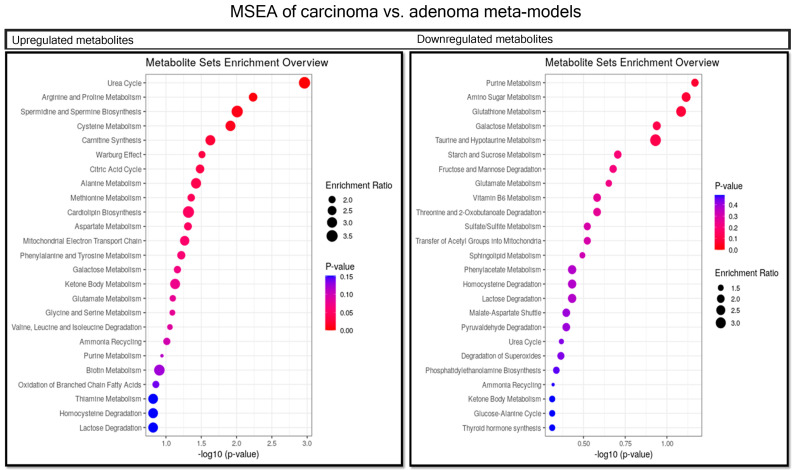
MSEA of the most important metabolites in PLS-DA model of carcinoma vs. adenoma. Metabolites more abundant in the carcinoma meta-model than the adenoma meta-model involved in pathways are shown in the left-side table. Those that are more abundant in adenomas are shown in the right-side table. We considered *p*-values < 0.05 significant for this analysis.

We used pairwise PLS-DA to find metabolic patterns among the meta-models of each group. PLS-DA is an efficient tool for analyzing metabolomic data [33]. It can effectively find patterns for differentiation between carcinoma vs. normal (Figure 3 and Figure 4), adenoma vs. normal (Figure 5 and Figure 6) and carcinoma vs. adenoma (Figure 7 and Figure 8) groups. In all the PLS-DA models’ first components, the most important metabolites that were involved in this differentiation were extracted from a VIP plot with a VIP criterion more than 1 [34,35]. We performed a cross-validation analysis of the PLS-DA models using the SIMCA software (which returns the significance of models). Supplementary File S5 describes the statistical parameters of the PLS-DA models in detail.

Metabolite set enrichment analysis (MSEA) was used to determine the role of metabolites in human cell metabolism. For this purpose, VIP was used to extract metabolites that were involved in human metabolism, filtered by the VMH database. This analysis was performed for metabolites that were increased or decreased in a pairwise analysis of groups. *p*-values < 0.05 were considered significant for the pathways detected in MSEA. Therefore, the results show that microbiota-derived metabolites could be involved in CRC metabolism by altering pathways under different conditions during CRC progression.

### 2.4. Comparison between Carcinoma and Normal Meta-Models

The results reveal that l-glutamine, l-tyrosine, pyruvate, tyramine, tryptamine and ten other metabolites were significantly increased in the normal meta-model. Furthermore, metabolites such as taurine, l-serine, chondroitin sulfate, mannose, putrescine and 73 other metabolites were decreased in this comparison (Figure 3).

The MSEA results indicated that thyroid hormone synthesis, purine metabolism, phenylalanine metabolism, the urea cycle, ammonia recycling, tyrosine metabolism and amino sugar metabolism pathways were enriched in the list of increased metabolites. The decreased metabolites were involved in spermidine and spermine biosynthesis, galactose metabolism, the urea cycle, taurine and hypotaurine metabolism and phosphatidyl ethanolamine biosynthesis (Figure 4).

### 2.5. Comparison between Adenoma and Normal Meta-Models

Histamine, spermidine, putrescin, hydrogen sulfide, L-tryptophan and 75 other compounds were the metabolites most significantly increased in the adenoma meta-model compared with the normal meta-model. Butyrate, phenol acetate, cobalt, taurine and 69 others were decreased (Figure 5).

As the MSEA demonstrated (Figure 6), the metabolites that decreased in the adenoma versus normal meta-models are those contributing to purine metabolism, the transfer of acetyl groups in mitochondrial pathways, the urea cycle and phosphatidyl ethanolamine biosynthesis. Furthermore, the metabolites increased are those contributing to the urea and TCA cycles; the metabolism of arginine, proline, phenylalanine, tyrosine and galactose; the biosynthesis of cardiolipin; and the mitochondrial electron transport chain. The biosynthesis of spermidine and spermine, unlike in the carcinoma meta-model, seem to be downregulated.

### 2.6. Comparison between Carcinoma and Adenoma Meta-Models

In the carcinoma versus adenoma meta-model comparison, we found shared and distinct metabolites that distinguish these tumor types. Adenine, d-glucose, l-aspartate, l-histidine, pyruvate and 32 other substances were substantially increased in the carcinoma meta-model. Additionally, acetaldehyde, trimethylamine, putrescine, ethanol, hydrogen sulfide and 85 others were significantly increased in the adenoma meta-model (Figure 7).

Furthermore, in the MSEA, cycles such as TCA and urea, and the metabolism of amino acids, such as arginine, proline, cysteine and alanine, were related to the increased metabolites. The synthesis of spermidine, spermine and carnitine, and the Warburg effect are related to these metabolites (Figure 8).

**Figure 9:**
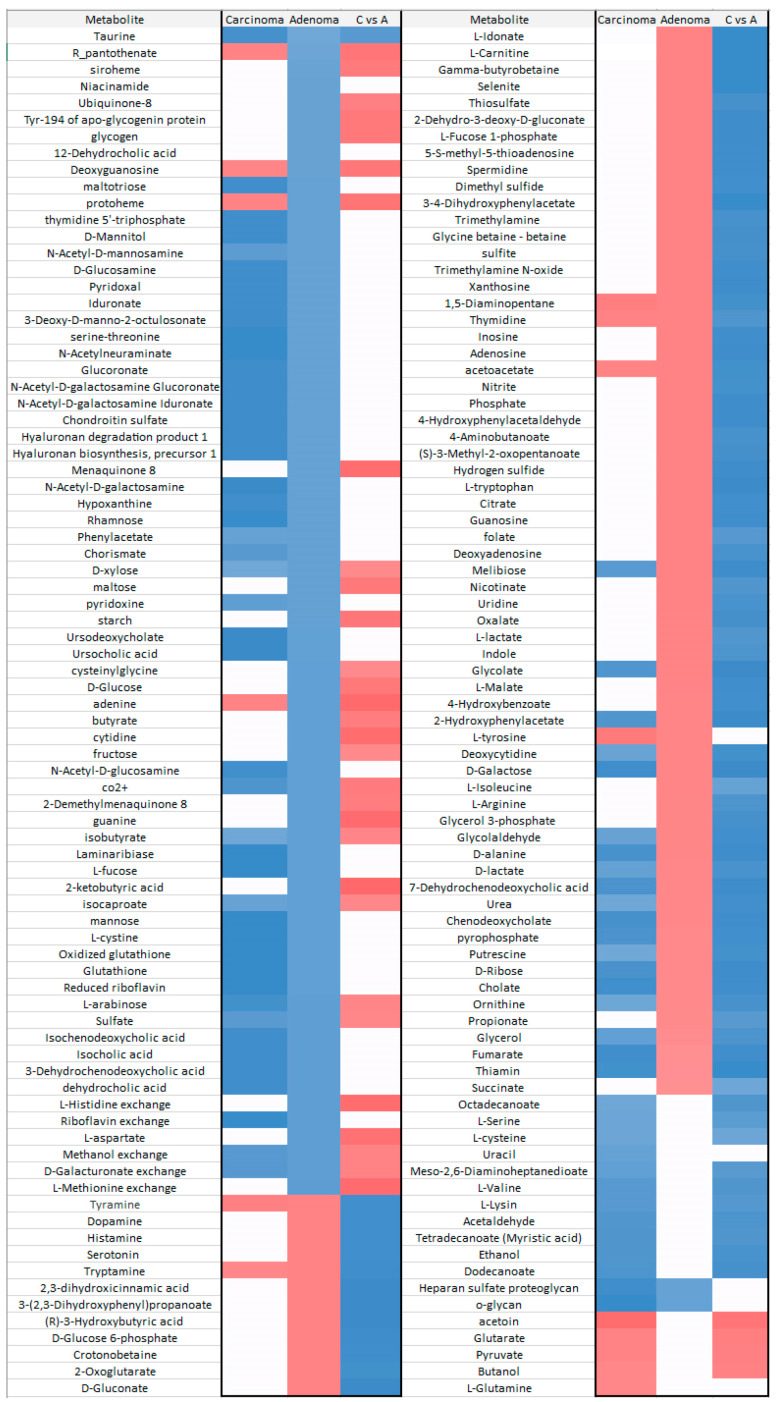
Significant metabolites in CRC microbiome. This heatmap briefly highlights significant metabolites altered in different tumors of CRC by our CMMs. Blue and red colors indicate decreases and increases in the intestinal lumen, respectively. White color means neither a significant alteration nor a contributor.

**Figure 10:**
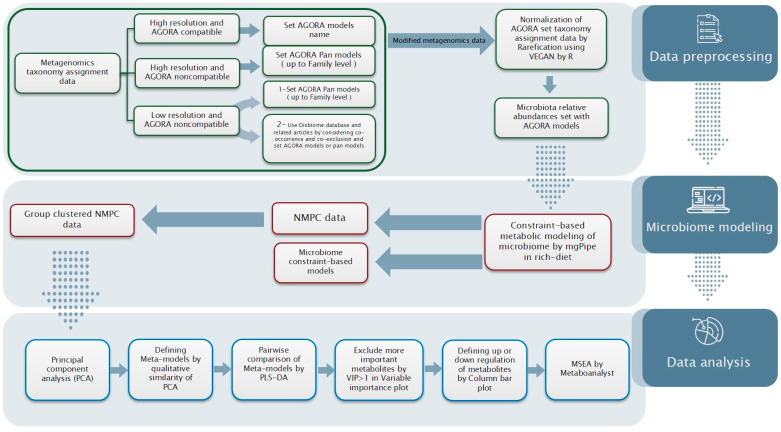
Proposed workflow for metabolic discrimination analysis of microbiome community constraint-based models, from data preparation to data analysis.

By the comparison of important metabolites between samples, we found that some of these metabolites are group-specific and some shared (Figure 9 and Figure 10). It showed that metabolites could be tumor-specific and have different roles in tumors. Notably, 37 metabolites significantly differentiated between normal, adenoma and carcinoma meta-models, and 13 specifically differentiated between two meta-models. As the driver–passenger hypothesis states, the metabolites of the CRC microbiota show different patterns in adenoma and carcinoma tumors. Some promote tumors and are released or remain and support tumor growth and development. Some metabolites only play a supportive role in carcinomas. On the other hand, the significant downregulation of certain metabolites, especially in the carcinoma meta-model, could be one reason for the detrimental effects of the carcinoma microbiota. As Figure 9 and Supplementary Figure S1 demonstrate, the carcinoma meta-model showed more downregulated than upregulated metabolites.

## 3. Discussion

Several studies have examined the association of metabolites with CRC. In vivo and in vitro environments cannot accurately indicate the sources of metabolic changes, especially in CRC, where the gut microbiome is an inseparable part. Based on the results of our microbiome community modeling and simulating the intestinal lumen metabolic environment (we have summarized the main results in Supplementary Figure S2), we hypothesized about how the microbiome composition affects CRC metabolism:

1. The adenoma microbiome plays an important role in the mutagenesis and the progression of the adenoma to carcinoma.
2. The metabolic changes in the adenoma microbiota increase inflammation and regulate the immune system.
3. The metabolites of the CRC microbiota contribute to the growth and proliferation of cancer cells in both adenoma and carcinoma tumors.
4. Microbial metabolites of adenomas and carcinomas are involved in the progression of CRC, for example, (the inhibition of) apoptosis and invasion.

Therefore, this workflow has the potential to investigate the underlying metabolic mechanisms regulating CRC progression, and it can be adopted to other disease microbial community models (Figure 10).

### 3.1. Adenoma Microbiota Plays an Important Role in Mutagenesis and Progression of Adenoma to Carcinoma

An increase in mutagenic metabolites and a decrease in their inhibitors involved in the progression of adenoma to carcinoma were observed in the metabolism of the adenoma microbiota. Short-chain fatty acids have many important functions within the human body, and numerous studies have shown that their levels significantly change in the fecal samples of patients with CRC [36]. Butyrate, one of the most important and controversial metabolites of the CRC microbiota, is significantly reduced in adenoma compared to healthy models. Butyrate is also involved in reducing inflammation and inducing apoptosis. In addition, it prevents the accumulation and formation of microbiota that cause epithelial cell mutations by regulating the immune system of the intestinal environment [37]. On the other hand, hydrogen sulfide (a toxic substance produced from the catabolism of meat foods) prevents the oxidation of butyrate and causes toxicity by disrupting the barrier of epithelial cells. It has been observed that these effects are more related to microbiota activity than diversity [37,38]. In our study, we showed that, in adenoma models, as H_2_S increases, ROS increase simultaneously. This is a point to consider in the bacterial flora’s role in pathogenicity, including CRC initiation.

Subsequently, other metabolites showed cooperation in CRC initiation. Adenoma models predicted an increase in spermidine and putrescine, which are polyamines. Polyamines play a critical role in mutagenesis and tumorigenesis by producing ROS [39]. The increased nitrite and electron transfer chain (ETC) activity in the MSEA indicates augmented ROS and mutagenicity. Nitrite acts as a precursor to N-nitroso compounds (NOCs) that are genotoxic and cause an increase in ROS [40]. Trimethylamines, including trimethylamine (TMA) and trimethylamine N-oxide (TMAO), are involved in DNA damage [41]. Increased urea can also increase ammonia production. Ammonia is involved in mutagenesis and tumorigenesis by damaging mucus, inducing genotoxicity and increasing ROS production. Increases in urea, TMA and TMAO are seen in adenoma models compared to healthy specimens [42].

Our models provide more evidence for the adenoma microbiota’s influence in CRC initiation by mutagenesis, although a VIP index greater than 1 was considered in the isolation of important metabolites. VIP values of 0.5 to 1 could also be significant [43]. Ethanol and acetaldehyde are among the substances whose mutagenic role in CRC has been discussed [37]. An increase in ethanol and acetaldehyde with a VIP of approximately 0.8 was seen in adenoma models. Increased tyramine was evident in both adenoma and carcinoma models. It is genotoxic to intestinal cells. It also further damages cancer cells by disrupting the DNA repair system [44]. In our opinion, despite the significant evidence in this section, there is still room for further investigation in future research.

### 3.2. Metabolic Alterations in the Adenoma Microbiota Increase Inflammation and Regulate the Immune System

Some metabolites play a key role by being multifunctional at different CRC levels. Hydrogen sulfide increases inflammation by reducing the oxidation of butyrate and breaking down the intestinal epithelial cell barrier. Furthermore, butyrate plays a role in reducing inflammation and the accumulation of harmful species by interacting with the immune system and producing a suitable environment [37]. Trimethylamines, in addition to inducing DNA damage, cause inflammation [37,41]. Increased hydrogen sulfide and trimethylamines and decreased butyrate in the adenoma microbiota meta-model, in addition to the role of mutagenicity, showed increasing inflammation.

Histamine is a chemical messenger made by immune cells that is also involved in inflammation. Previous studies have shown increased production of histamine in CRC and decreased catabolism in CRC adenomas. It may also play a role in the development of CRC by affecting the histamine 2 receptor (H2R) [45]. Although the production of this substance has not been studied in previous studies from the perspective of the microbiota and the role of this organ, in the adenoma meta-model, histamine production was significantly increased. Simultaneous reductions in chondroitin sulfate and glucosamine were observed in both the adenoma and carcinoma meta-models. The possible effects of these two substances on the initial prevention of CRC, with anti-inflammatory properties, were previously investigated [46].

### 3.3. CRC Microbiota Contribute to the Growth and Proliferation of Cancer Cells in Both Tumors

The complete urea cycle converts excess nitrogen to urea and excretes it in the urine. This complete cycle is mainly active in the liver, but enzymes in other cells are responsible for the synthesis of the intermediates of this cycle through the use of nitrogen according to cells’ needs. Extrahepatic urea cycle enzymes are the only intracellular producers of arginine, citrulline and ornithine, which are precursors for the synthesis of polyamines, nitric oxide (NO) and proline. In cancer, unlike in liver cells that secrete nitrogen, urea cycle mediators, including arginine, proline and ornithine, enter anabolic pathways, and changes in their enzymes contribute to tumor growth [42]. As ammonia production increases, ammonia condensation with bicarbonate produces carbamoyl phosphate (CP) through the enzyme carbamoyl phosphate synthase (CPS1), which prevents ammonia toxicity. It has been observed that, in cancer, the CP barrier between the mitochondria and the cytoplasm disappears, and cytoplasmic CP increases [47,48]. Finally, the CAD protein (a trifunctional multi-domain enzyme including carbamoyl phosphate synthase 2, aspartate trans-carbamylase and dihydro-orotase) converts cytoplasmic CP to pyrimidines, which are required for cell proliferation. Furthermore, increased CPS1 expression has been observed to be associated with a poor prognosis in CRC [49,50]. In the MSEA, the urea cycle was increased in the adenoma and carcinoma meta-models, and it was shown that the CRC microbiota may play a key role in increasing the activity of this cycle in human cells.

Interestingly, arginine, proline and ornithine, which are important in the urea cycle, were increased in the adenoma models. Furthermore, the increase in arginine and proline metabolism in human cells was increased according to the MSEA results. Arginine and proline replace glucose in the energy supply under glucose deprivation and energy deficiency. The enzyme arginine succinate lyase (ASL) is highly expressed in various cancers, including CRC, which produces arginine, NO and citrulline. Furthermore, the production of intracellular NO from arginine is dependent on this enzyme, so the inhibition of this enzyme has similar effects on the reduction of NO. NO promotes cell proliferation [42].

The amount of secreted glutamine, an important hallmark metabolite of cancer metabolism, was increased in the carcinoma meta-model. Cancer cells use glutamine during glutaminolysis for cell growth and proliferation. This is one of the hallmarks of cancers, and many treatment strategies have been developed based on it. Cancer cells use mediators created in the glutaminolysis cycle to replenish the TCA cycle [51]. Glutamine is probably needed for tumors to become malignant. By producing ammonia, glutamine regulates the intercellular physiological pH of cancer cells as a buffer [52]. In addition to glutamine, pyruvate was further increased in the carcinoma meta-model.

Pyruvate is present in anaplerotic reactions in ovarian cancer and affects mitochondrial functions [53]. By affecting the ETC, it causes the production of ROS and alters cell proliferation [54]. The serine racemase enzyme produces pyruvate from serine. The role of this enzyme in CRC becomes more prominent with the production of pyruvate, and it aids tumor growth and is being investigated as a drug target [55]. Interestingly, our models confirmed these findings: firstly, its gene is present in Firmicutes, Actinobacteria and Fusobacteria, which are present and prevalent in CRC microbiota models. Secondly, there were decreased levels of serine and increased levels of pyruvate in the carcinoma meta-model and, thirdly, there is increased pyruvate uptake from the environment in some cancers. Finally, the increase in pyruvate demonstrated its supportive role as an energy supplier and in increasing cell proliferation in the carcinoma meta-model. According to the results of our modeling, the influence of the microbiome in this regard can be very significant.

Despite the principle of ignoring the TCA cycle and the priority of aerobic glycolysis in cancer cell metabolism, the TCA cycle was increased in the MSEA of the adenoma meta-model. The TCA is involved in the production of required macromolecules and the energy production of cancer cells [51]. From these results, we could deduce that the microbiome contributed to an increase in the metabolic cycle, energy production and cancerous tumor formation.

The effects of hormones on pathogenicity and its origin are usually considered in the physiology of a human body. The MSEA results revealed the enrichment of thyroid hormone synthesis in the carcinoma meta-model. There have been many studies on the effects of thyroid hormone and nuclear receptors on tumorigenesis and cancer cell proliferation. Despite the heterogeneity in these studies, increased receptor expression in CRC and increased risk in patients with hyperthyroidism may be of interest. It should also be noted that, given the environmental uptake of cancer cells, the role of microbiome metabolism in the production and explanation of the effect of this hormone could be important [56,57].

Serotonin and dopamine were also increased in the adenoma meta-model. These are neurotransmitters produced by the central nervous system and the gastrointestinal tract. Clinical studies have proven their effects on the growth of tumors such as CRC [58,59,60,61,62,63,64]. Furthermore, the overexpression of the serotonin B2 receptor, which leads to increased cell proliferation, is potential evidence of the effect of serotonin on CRC cell proliferation [65]. These roles in CRC were also proved by our models, but with the collaboration of the microbiota.

Other pathway alterations involved cardiolipin and ETC biosynthesis, which were enriched in the MSEA results for the adenoma meta-model. Cardiolipin is present in the inner membrane of the mitochondria; its overexpression and interaction with ETC proteins in CRC cells increases and optimizes the efficiency of mitochondrial respiration [66]. The inhibition of the ETC also reduces tumor growth [67]. Considering the role of cardiolipin in membrane structure and its interaction with ETC, the effect of adenoma microbiome of increasing cell proliferation through supporting mitochondrial precursors and energy supply through the ETC has been demonstrated.

The gut microbiome can also accelerate the production of purine and pyrimidine precursors, supporting cell proliferation and energy supply. Our results show significant increases in nucleotides, DNA precursors and purine metabolism in the adenoma and carcinoma models. Adenine, adenosine and deoxy-adenosine, deoxy-cytidine, uridine, guanosine and thymidine were found among the enhanced metabolites for both models. Thymidine catabolism is a metabolic strategy of cancer cells that provides them with energy by supplying carbon to the glycolytic pathway under nutritional deprivation [68].

Phenolic acids and cobalt chloride have anticancer effects [69,70]. The decrease in phenolic acids and cobalt in cancer models compared to healthy individuals is in good agreement with the role of the microbiome in reducing cell proliferation.

### 3.4. Microbiome Metabolites in Adenoma and Carcinoma Are Involved in the Development of Colorectal Cancer, such as through (the Inhibition of) Apoptosis and Invasion

Our carcinoma meta-data model indicated the prevention of apoptosis due to a significant reduction in the synthesis of taurine, spermidine, phosphatidylethanolamine and phenolic acid metabolites. Taurine stimulates apoptosis, and its secretion in the carcinoma microbiome may be reduced [71,72]. Spermidine, which was increased in the adenoma model and was involved in inflammation and tumorigenesis, was decreased in the carcinoma meta-model. The dual role of spermidine in cancer was previously investigated. Spermidine can play a role in tumor suppression by increasing apoptosis and autophagy, and decreasing immunosuppression [39]. The reduction of this metabolite in the carcinoma meta-model showed the role of the microbiome in inhibiting the anticancer effect of spermidine. The role of phosphatidylethanolamine in stimulating apoptosis by reducing the mitochondrial membrane potential in hepatocytes has been previously demonstrated [73]. The decreased synthesis of this substance in the carcinoma microbiome probably prevents apoptosis. Decreases in galacturonate and butyrate metabolites were seen in the adenoma meta-model. Galacturonate-containing pectins have been introduced as drugs that increase apoptosis, as well as carriers, and in combination with anticancer drugs [74]. A decrease in butyrate, a substance that induces apoptosis, prevents cancer cell apoptosis [75,76]. The elevated glutamine and pyruvate in the carcinoma meta-model showed that the microbiome is able to provoke malignancy and cancer migration. Pyruvate has previously been shown to support the migration and development of ovarian cancer [53]. The presence of glutamine may also be essential for cancer malignancy [52]. Increased arginine and, eventually, higher production of NO, dopamine and serotonin, indicated the potential for the microbiome to affect cancer cell angiogenesis. NO can be involved in epithelial-to-mesenchymal transition (EMT) and angiogenesis [42]. Clinical studies have shown the effects of serotonin and dopamine on the angiogenesis of cancers such as CRC [65]. Fumarate affects the migration and invasion of cancer cells by acting on the killer cell lectin-like receptor C3. An increase in this metabolite in the adenoma meta-model could confirm this effectiveness [77].

## 4. Materials and Methods

In this study, we investigated microbiome metabolism in terms of the abundances of microbiota and their distinct metabolomics in different tumors of CRC. For this purpose, we used metabolic modeling and metabolomics analysis approaches including community metabolic modeling (CMM), principal component analysis (PCA) and metabolite set enrichment analysis (MSEA). We used metagenomics and the relative abundances of microbiota data as the input for microbiome metabolic modeling. Figure 10 depicts the workflow used for this study. To determine the compatibility of the input data with the methodology, we preprocessed the metagenomic data.

### 4.1. Data Collection and Preprocessing

#### 4.1.1. Taxonomy Assignment Data

The relative abundances of microbiota in different tumors of CRC were extracted from the MGnify database (study MGYS00001248). The dataset consists of mucus biopsies from 160 individuals harvested by colonoscopy and examined histologically [21], and 61 tumor-free specimens, 47 patients with adenoma polyps and 52 carcinoma patients.

#### 4.1.2. Data Preprocessing

To create a CMM, we require (1) the relative abundances of presented and recognized bacteria, and (2) individual GEMs that match the recognized bacteria. For data consistency, we manually modified the taxonomy nomenclature because metagenomic data often do not have sufficient resolution at the strain, species and genus levels for the bacteria. For the compatibility of the data with the taxonomy assignments, high-resolution taxon data were matched with AGORA GEM models derived from the Virtual Human Metabolism [78] (VMH) database. For non-compatible data, we used the “createPanModels” function in the Constraint-Based Reconstruction Analysis (COBRA) toolbox [79] to construct Pan models. A Pan model is a GEM constructed from a combination of some other GEMs.

To modify the taxonomy nomenclature, we used the Disbiome database [28]. This database presents the dysbiosis of the microbiome in diseases, and the nearest strain, species, or genus affected by CRC according to its decrease or increase in abundance. Taxonomy levels higher than family were matched to AGORA models and Pan models by a literature review and using the Disbiome database.

Data normalization is challenging in this domain. One of the most applicable methods is rarefaction, the applications of which in metagenomics and microbiome analysis have been discussed [29,30,31]. The average number of sample read counts was about 10,000. To confirm this value, we also plotted the rarefaction curve (Figure 1) and considered its asymptote. Curve plotting and data normalization were performed using the rarefaction method in the Vegan [80] library in R.

### 4.2. Microbiome Metabolic Modeling

Large-scale community modeling requires the integration of the relative abundances of metagenomics data into metabolic models. For this purpose, we used the mgPipe pipeline that is the part of the microbiome modeling toolbox in the COBRA toolbox. The mgPipe function includes: (1) the analysis of the microbiota abundances per sample; (2) the construction of microbiome models and adding lumen-transport reactions by connection to a specific diet and uptake/secretion behavior; and (3) the simulation of models and the lumen metabolic environment under the given diet by flux variable analysis (FVA). These simulations demonstrate the maximal capability for metabolite production in a lumen environment: the so-called net maximal production capacity (NMPC). The metabolic modeling of the microbiome community was performed using the COBRA toolbox in the MATLAB 2017b environment with the IBM CPLEX 12.8 solver. We used the mgPipe pipeline and AGORA 1.03 [26] models and their Pan models to reconstruct CMM on a rich diet. In fact, the rich diet supports the stability of the relative abundances of bacteria in an environment without limitations in nutrient-uptake fluxes. Therefore, we selected the rich diet condition for further modeling.

### 4.3. Data Analysis

#### 4.3.1. Multivariate Analysis

NMPC data show the maximum microbiota community capacity of each sample in creating the intestinal lumen metabolic environment. This information was used to analyze and compare within groups and between groups.

PCA and partial least-squares discriminant analysis (PLS-DA) are two methods that are important in the analysis of metabolomic data [33]. In this study, due to the high number of features, we first ran PCA to investigate the similarity and discrimination between all the samples and to separate similar models of each group—the so-called meta-model. Then, PLS-DA was performed for comparison between groups.

The variable important projection (VIP) used PLS-DA information to display each of the variables in the first component of each PLS-DA model.

The PCA and PLS-DA were performed in the SIMCA software environment of UMETRICS company version 14.1. Autofit was performed for PCA and PLS-DA modeling with default settings.

The cross validation of the PCA and PLS-DA was performed using the SIMCA software, by full cross validation. The method used the Krzanowski and PRediction Error Sum of Squares (PRESS) methods and returned the significance of the models [81,82].

#### 4.3.2. Metabolic Set Enrichment Analysis

MSEA [83] is an approach to identifying and interpreting patterns in human metabolism through metabolic alterations according to metabolic data. The VMH database was used to isolate microbiota-produced metabolites involved in human metabolism from all the metabolites derived from the PLS-DA and then VIP extraction. These metabolites were used as input for the Metaboanalyst platform [84].

## 5. Conclusions

Our community metabolic models unveiled the roles of the gut microbiome in CRC development, as well as its significant influence on adenoma and carcinoma tumors, from provoking mutations to facilitating the spread, homeostasis and survival of colorectal cancer cells.

## Figures and Tables

**Table 1:** Numbers of samples at different steps of this study. Adjacent tissue samples in MGYS00001248 data were discarded since we wanted to focus on in situ tumor and polyp microbiomes. In the normalization step, samples with low read counts were removed (in total, 76 of 170 samples were removed). At the modeling step, due to unknown errors, some models were infeasible, which we excluded from further analysis. Finally, we considered microbiome metabolism profiles clustered as meta-models.

	Normalization Input	Normalized Input for Microbiome Metabolic Modeling	Reconstructed Microbiome Metabolic Models	Data Analysis and Meta-Model Selection
Adenoma	57	41	37	8
Carcinoma	52	26	24	7
Normal	61	27	27	6
Total	170	94	88	21

## Data Availability

All the data are available at https://www.ebi.ac.uk/metagenomics/search?query=MGYS00001248 (accessed on 23 September 2020).

## Supplementary Materials

The following are available online at https://www.mdpi.com/article/10.3390/metabo11070456/s1. Supplementary File S1: Pan AGORA models (upon request), Supplementary File S2: Bacterial GEMs’ names and their abundances for further analysis, Supplementary File S3: Microbiome community models (upon request), Supplementary File S4: Results for net maximal production capability, Supplementary File S5: Statistical results of PCA and PLS-DA, Figure S1: Venn diagram of VIP > 1 metabolites. Figure S2: Hypothesized about how the microbiome composition affects CRC metabolism.
