# Supplementary figures and images for: A Metabolic Model of Intestinal Secretions: The Link between Human Microbiota and Colorectal Cancer Progression
Source: Metabolites. 2021 Jul 15;11(7):456. doi: 10.3390/metabo11070456 (PMC8303431; doi:10.3390/metabo11070456)

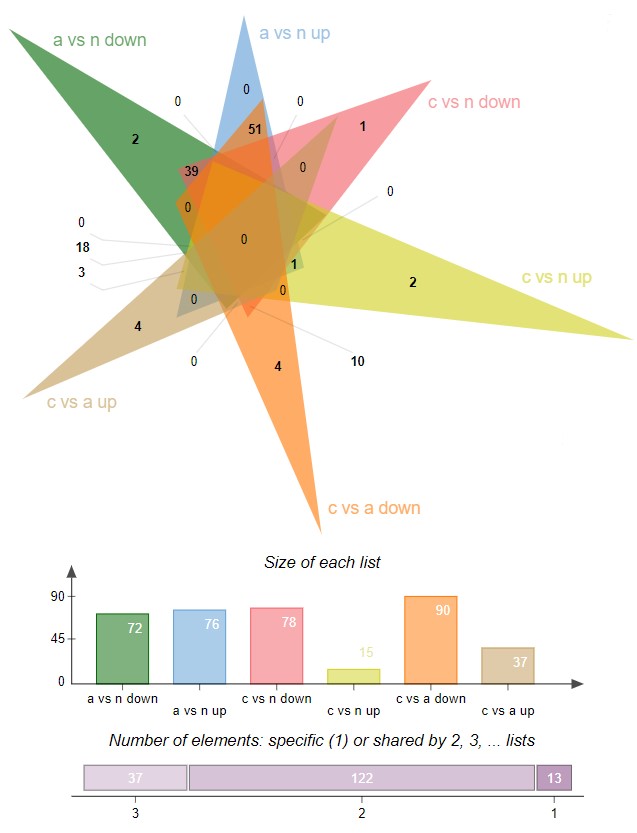

Supplement: Supplementary file 1 [file metabolites-11-00456-s001.zip › Supp/Supplementary figure 1.jpg]

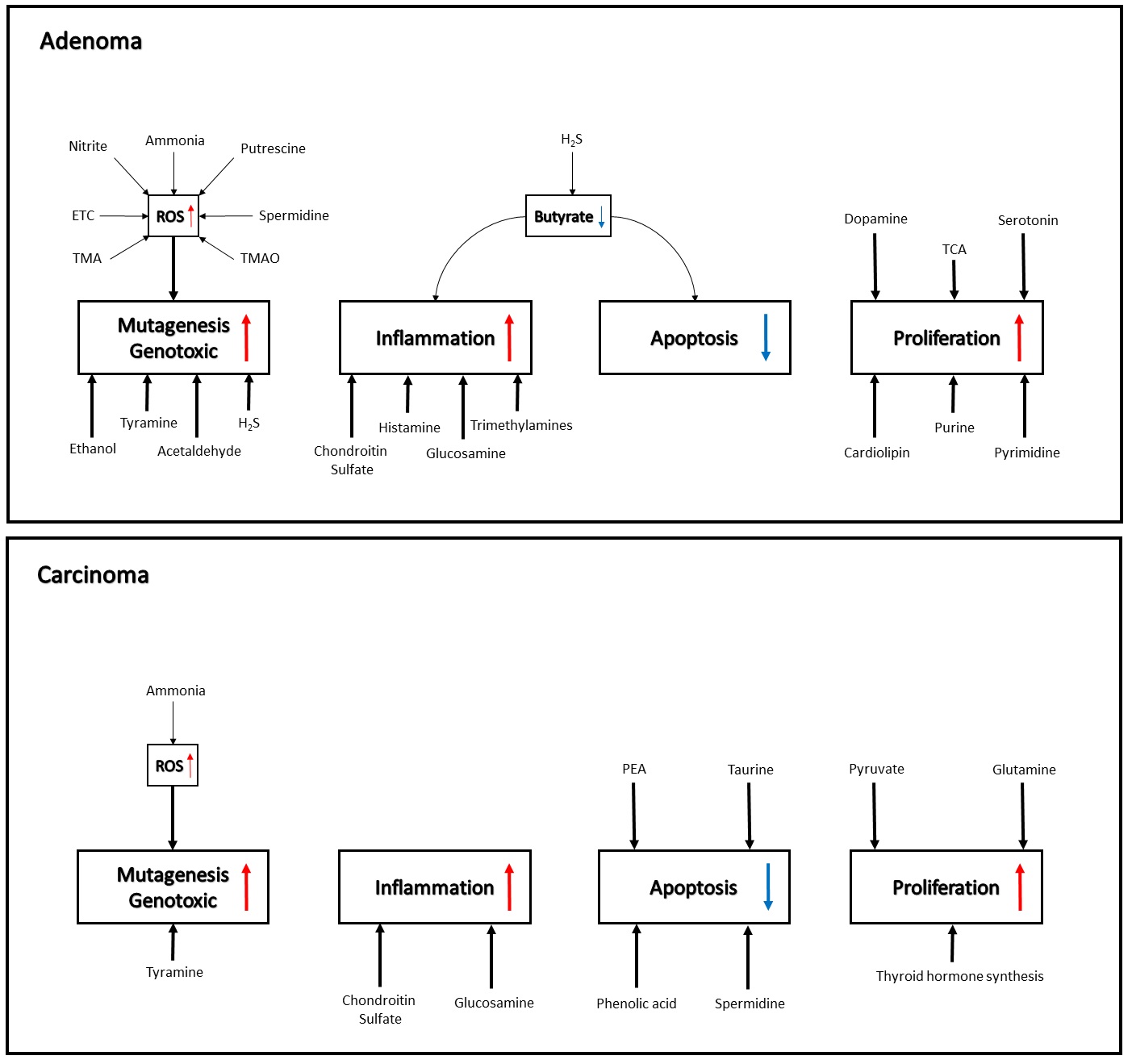

Supplement: Supplementary file 1 [file metabolites-11-00456-s001.zip › Supp/Supplementary figure 2.jpg]
